# Association between C-reactive protein/albumin ratio and mortality in older Japanese patients with post-stroke dysphagia

**DOI:** 10.3389/fneur.2025.1648517

**Published:** 2025-11-21

**Authors:** Ziying Wang, Lingling Wang

**Affiliations:** 1 Department of Postgraduate, School of Clinical Medicine, Beihua University, Jilin, China; 2 Stroke Unit, Affiliated Hospital of Beihua University, Jilin, China

**Keywords:** C-reactive protein/albumin ratio, dysphagia, elderly population, mortality, stroke

## Abstract

**Introduction:**

The C-reactive protein/albumin ratio (CAR) could serve as an novel obtainable application for prognosis. Few studies have assessed CAR and mortality in older Japanese patients with post-stroke dysphagia (PSD). We evaluated CAR as a composite prognostic factor and compared its predictive performance with C-reactive protein (CRP) and albumin.

**Methods:**

In this retrospective cohort study, 133 elderly Japanese patients with PSD (January 2014–January 2017) were analyzed. Cox proportional hazards models estimated associations of CAR, CRP, and albumin with mortality. Survival across CAR tertiles was compared by Kaplan–Meier curves. For 1-year mortality prediction we computed ROC curves and assessed incremental value using IDI and NRI.

**Results:**

Among 133 patients (78 women, 55 men; mean age 80.5 years), each one-unit increase in CAR was associated with a 42% higher hazard of death in unadjusted analysis (HR 1.42; 95% CI 1.18–1.71; *p* < 0.001) and remained significant after full adjustment (HR 1.49; 95% CI 1.16–1.92; *p* = 0.002). Higher CAR tertiles had worse survival (*p* < 0.0001). For 1-year mortality discrimination, CAR AUC = 0.702 (95% CI 0.606–0.798), albumin AUC = 0.710, CRP AUC = 0.685. Albumin improved prediction over CAR (IDI 0.108, *p* = 0.012; NRI 0.217, *p* = 0.044); CRP did not.

**Conclusion:**

CAR was positively associated with mortality in older Japanese patients with PSD but had only modest discrimination for 1-year mortality. Albumin provided a small additional predictive benefit. Larger prospective studies are needed for validation.

## 1 Introduction

C-reactive protein (CRP) is an inflammatory application produced by the liver in response to interleukin-6 (1). Albumin is a principal indicator of nutritional status and also modulates inflammatory responses (2). Because CRP and albumin reflect, respectively, systemic inflammation and nutritional reserve, the C-reactive protein/albumin ratio (CAR) has emerged as a composite application that captures the balance between inflammation and nutrition. CAR has demonstrated prognostic value across a range of conditions, including malignant tumors (3), critical illnesses (4), hematological diseases (5), orthopedic infections (6) and metabolic disorders (7).

Post-stroke dysphagia (PSD) is a common, clinically important complication of stroke and is associated with adverse outcomes (8). Epidemiological estimates indicate that 37–78% of stroke patients develop dysphagia, with prevalence depending on screening methods (9). Among stroke survivors, 60.9% report at least one type of eating difficulty. Neurogenic dysphagia commonly results in atrophy of the swallowing musculature and raises the risk of aspiration pneumonia, which can impede recovery (8). PSD is frequently accompanied by heightened inflammatory activity and deteriorating nutritional status (10). An elevated CAR reflects this combined “inflammation-nutrition imbalance.” That imbalance may worsen swallowing function and clinical prognosis. Prior studies have linked CAR to poor outcomes in acute stroke patients (11), but its prognostic value specifically in chronic PSD is unclear.

We focus on older Japanese patients because Japan has the world’s most rapidly aging population (12), making PSD and related complications especially consequential. Therefore, this study evaluated the association between CAR and mortality in older Japanese patients with PSD using available clinical and laboratory data. We hypothesized that higher CAR would be associated with increased mortality in this cohort.

## 2 Methods

### 2.1 Study design and participants

This retrospective observational study included elderly patients diagnosed with PSD between January 2014 and January 2017. Included patients received either percutaneous endoscopic gastrostomy (PEG) or total parenteral nutrition (TPN). The severity of dysphagia was confirmed through comprehensive clinical assessment by a multidisciplinary team (doctors, nurses, and speech therapists) and video fluoroscopic swallowing studies. All the enrolled subjects had a history of stroke, as recorded in their medical records. Exclusion criteria included patients with advanced cancer, those who needed PEG for gastric decompression, those who had received PEG before January 2014, and those without a history of stroke. This study was approved by the Institutional Review Board of the Miyomori Memorial Hospital. As the data were anonymized and met the regulatory standards, the requirement for informed consent was waived.

### 2.2 Data source

Data for this study were obtained from the Dryad Digital Repository, which provides unrestricted access to the original dataset. According to Dryad’s guidelines, we cited the data package titled “Baseline C-reactive protein, albumin level, and life prognosis in Dryad,” which can be accessed via https://doi.org/10.5061/dryad.gg407h1. These findings were published in 2019 (13). The original study compared outcomes of PEG versus TPN. In contrast, the present analysis focuses on CAR as a novel application. This difference in focus differentiates our work from the original publication and prevents duplication of its aims.

### 2.3 Procedures

The primary endpoint was all-cause mortality during the follow-up period. Nutritional intervention protocols were individualized based on multidisciplinary assessments. Demographics (age, sex, body mass index [BMI]), comorbidities (severe dementia, ischemic heart disease, and aspiration pneumonia), Clinical Frailty Scale (CFS) score, oral intake recovery status, and laboratory measures were abstracted from medical records. Hematological parameters were collected within 1 week prior to initiation of nutritional intervention. CAR was calculated using the following formula. Participants were categorized into tertiles for CAR, CRP and albumin as follows: CAR T1 < 0.093, T2 0.093–0.604, T3 ≥ 0.604; CRP T1 < 0.32 mg/dL, T2 0.32–1.81 mg/dL, T3 ≥ 1.81 mg/dL; albumin T1 < 3.0 g/dL, T2 3.0–3.6 g/dL, T3 ≥ 3.6 g/dL.


CAR=CRP/Albumin

### 2.4 Statistical analysis

Categorical variables were presented as percentages, whereas continuous variables are presented as mean±SD or median (IQR). Baseline characteristic comparisons were performed using one-way ANOVA for continuous variables and chi-square or Fisher’s exact tests for categorical variables. We use Cox proportional hazards regression to estimate hazard ratios (HRs) and 95% confidence intervals (CIs) for associations between CAR, CRP, albumin, and mortality. The proportional hazards assumption was assessed by Schoenfeld residuals and met for the outcome of death. We constructed three Cox models: Model 1 unadjusted; Model 2 adjusted for age, sex and BMI; Model 3 additionally adjusted for CFS score, oral intake recovery and aspiration pneumonia. Kaplan–Meier curves with log-rank tests compared survival across tertiles of CAR, CRP and albumin. Predictive performance for 1-year mortality was evaluated by ROC curves and AUCs with 95% CIs. Incremental prognostic value of CRP and albumin over CAR was assessed using integrated discrimination improvement (IDI) and net reclassification improvement (NRI). The interaction effects were examined using likelihood ratio tests. Sensitivity analyses included alternative CAR cutoff, comparison across four model specifications, and calculation of the E-value to quantify robustness to unmeasured confounding. To determine whether the association between CAR and mortality was attributable to hypoalbuminemia, we stratified the cohort by serum albumin into two groups. The high-albumin group had albumin ≥ 3.6 g/dL. The low-albumin group had albumin < 3.6 g/dL. Within each group, we used Cox proportional hazards regression to assess associations between CAR, CRP, albumin and mortality. We reported HRs and 95% CIs. Model 1 was unadjusted. Model 2 adjusted for age, sex and BMI. Analysis was performed using R 4.2.2 (http://www.Rproject.org; The R Foundation, Vienna, Austria) and the Free Statistics software (version 2.1.1; Beijing FreeClinical Medical Technology Co., Ltd., Beijing, China).

## 3 Results

### 3.1 Study participants and baseline characteristics

Table 1 shows the key characteristics of the cohort of 133 patients (55 males and 78 females). Because age followed a normal distribution, it was reported as mean±SD (80.5 ± 10.4 years). Among these patients, 107 underwent PEG and 26 underwent TPN. Tertile comparisons identified significant differences in sex, PEG, CFS score, ischemic heart disease, severe dementia, CRP and albumin (*p* < 0.05). Patients in the highest CAR tertile had higher prevalences of severe dementia and ischemic heart disease and were more frequently managed with TPN.

**Table 1:** Baseline characteristics of patients.

Variables	Total	CAR			*p*
		T1 (<0.093)	T2 (0.093 ~ 0.604)	T3 (≥0.604)	
Age (years), Mean ± SD	80.5 ± 10.4	79.0 ± 10.2	79.4 ± 12.6	83.2 ± 7.7	0.113
Sex, *n* (%)					0.009
Male	55 (41.4)	10 (22.7)	23 (52.3)	22 (48.9)	
Female	78 (58.6)	34 (77.3)	21 (47.7)	23 (51.1)	
BMI (kg/m^2^), Mean ± SD	19.7 ± 3.4	19.6 ± 2.4	20.3 ± 4.1	19.1 ± 3.5	0.242
PEG, *n* (%)	107 (80.5)	41 (93.2)	39 (88.6)	27 (60)	< 0.001
Oral intake recovery, *n* (%)	9 (6.8)	5 (11.4)	3 (6.8)	1 (2.2)	0.194
Aspiration pneumonia, *n* (%)	32 (24.1)	5 (11.4)	14 (31.8)	13 (28.9)	0.052
CFS score, *n* (%)					0.035
4	1 (0.8)	1 (2.3)	0 (0)	0 (0)	
7	17 (12.8)	5 (11.4)	10 (22.7)	2 (4.4)	
8	115 (86.5)	38 (86.4)	34 (77.3)	43 (95.6)	
Ischemic heart disease, *n* (%)	30 (22.6)	4 (9.1)	9 (20.5)	17 (37.8)	0.005
Severe dementia, *n* (%)	17 (12.8)	1 (2.3)	5 (11.4)	11 (24.4)	0.007
CRP (mg/dl), Median (IQR)	0.8 (0.2, 2.4)	0.2 (0.1, 0.2)	0.8 (0.5, 1.1)	4.0 (2.4, 6.0)	<0.001
Albumin (g/dl), Mean ± SD	3.3 ± 0.6	3.7 ± 0.4	3.3 ± 0.5	2.8 ± 0.6	<0.001

BMI, body mass index; CAR, C-reactive protein/albumin ratio; CRP, C-reactive protein; CFS, Clinical Frailty Scale scores; CFS score were grouped as 4, 7, and 8 to indicate severity; PEG, percutaneous endoscopic gastrostomy.

### 3.2 Association between CAR, CRP, albumin and mortality

In Cox regression analyses (Table 2), CAR, CRP and albumin were significantly associated with mortality. We constructed three Cox models. Model 1 was unadjusted. Model 2 was adjusted for age, sex and BMI. Model 3 was additionally adjusted for CFS score, oral intake recovery and aspiration pneumonia. In the fully adjusted model (Model 3), continuous CAR was associated with higher mortality (HR 1.49, *p* = 0.002), as was continuous CRP (HR 1.15, *p* = 0.008); continuous albumin was associated with lower mortality (HR 0.35, *p* < 0.001). Compared with the lowest tertile, the highest CAR tertile conferred a 3.77-fold increased mortality risk, and the highest CRP tertile a 3.92-fold increased risk; albumin ≥3.6 g/dL was associated with a 75% reduction in mortality risk. Trend tests across tertiles were significant.

**Table 2:** Association between CAR, CRP, albumin and mortality in different models.

Variable	Model 1		Model 2		Model 3	
	HR (95%CI)	*p*	HR (95%CI)	*p*	HR (95%CI)	*p*
CAR (continuous)	1.42 (1.18 ~ 1.71)	<0.001	1.39 (1.12 ~ 1.72)	0.003	1.49 (1.16 ~ 1.92)	0.002
CAR (T1, <0.093)	1(Ref)		1(Ref)		1(Ref)	
CAR (T2,0.093 ~ 0.604)	2.91 (1.29 ~ 6.57)	0.010	2.19 (0.95 ~ 5.05)	0.066	2.55 (1.09 ~ 5.95)	0.031
CAR (T3, ≥0.604)	5.64 (2.60 ~ 12.27)	<0.001	3.88 (1.75 ~ 8.62)	0.001	3.77 (1.70 ~ 8.35)	0.001
*P* for trend		<0.001		<0.001		0.001
CRP (continuous)	1.14 (1.05 ~ 1.23)	0.001	1.12 (1.03 ~ 1.22)	0.010	1.15 (1.04 ~ 1.27)	0.008
CRP (T1, <0.32)	1(Ref)		1(Ref)		1(Ref)	
CRP (T2,0.32 ~ 1.81)	3.3 (1.47 ~ 7.43)	0.004	2.79 (1.23 ~ 6.34)	0.014	3.17 (1.39 ~ 7.27)	0.006
CRP (T3, ≥1.81)	5.1 (2.34 ~ 11.11)	<0.001	3.88 (1.75 ~ 8.59)	0.001	3.92 (1.77 ~ 8.67)	0.001
*P* for trend		<0.001		0.001		0.001
Albumin (continuous)	0.32 (0.21 ~ 0.48)	<0.001	0.37 (0.23 ~ 0.59)	<0.001	0.35 (0.22 ~ 0.56)	<0.001
Albumin (T1, <3.0)	1(Ref)		1(Ref)		1(Ref)	
Albumin (T2,3.0 ~ 3.6)	0.47 (0.27 ~ 0.82)	0.008	0.44 (0.24 ~ 0.79)	0.006	0.39 (0.21 ~ 0.71)	0.002
Albumin (T3, ≥3.6)	0.18 (0.09 ~ 0.36)	<0.001	0.28 (0.13 ~ 0.62)	0.002	0.25 (0.11 ~ 0.56)	0.001
*P* for trend		<0.001		0.001		<0.001

Model 1: Non-adjusted. Model 2: Adjusted for age, sex and BMI. Model 3: Adjusted for age, sex, BMI, Clinical Frailty Scale scores, oral intake recovery and aspiration pneumonia. BMI, body mass index; CAR, C-reactive protein/albumin ratio. CRP, C-reactive protein.

**Figure 1:**
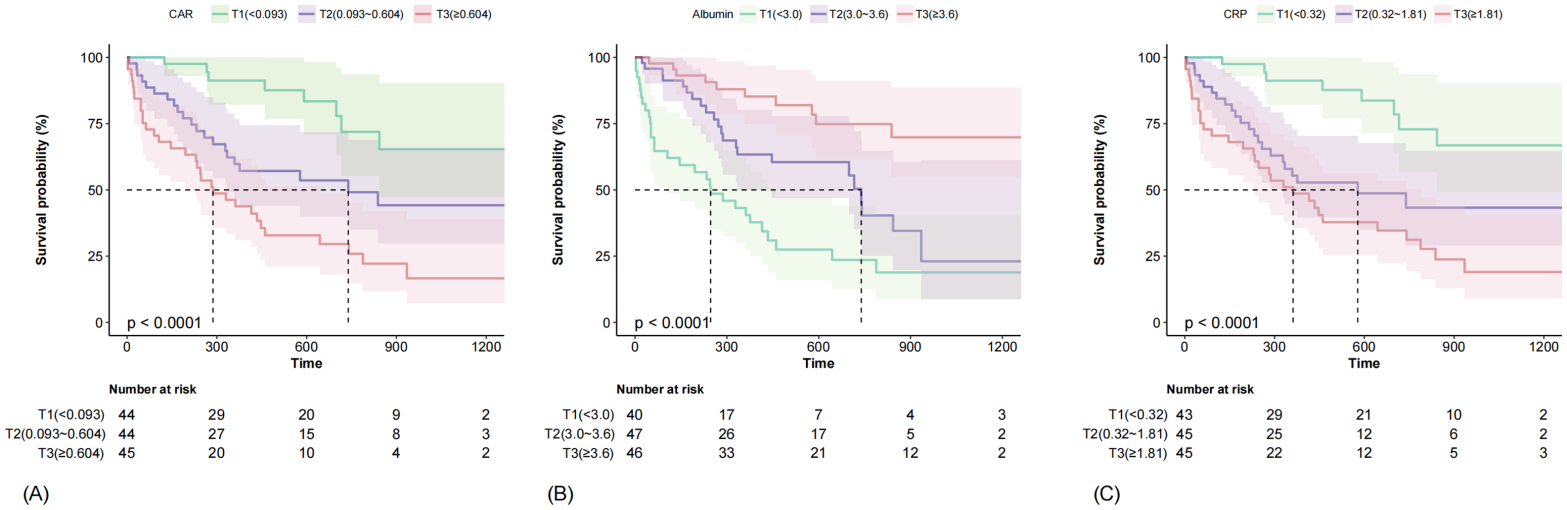
Kaplan–Meier survival analysis for all-cause mortality stratified by tertiles of CAR **(A)**. Kaplan–Meier survival analysis for all-cause mortality stratified by tertiles of albumin **(B)**. Kaplan–Meier survival analysis for all-cause mortality stratified by tertiles of CRP **(C)**. CAR, C-reactive protein/albumin ratio; CRP, C-reactive protein.

Kaplan–Meier curves demonstrated significant differences in mortality across tertiles of CAR, CRP and albumin (all *p* < 0.0001, Figure 1). For CAR, median survival was 282 days in T3 and 762 days in T2, while median survival in T1 was not reached (maximum follow-up 1,463 days). For CRP, median survival was 330 days in T3 and 658 days in T2, with T1 not reaching median survival. For albumin, the T1 had median survival of 234 days, T2 had 716 days, and median survival in T3 was not reached.

**Figure 2:**
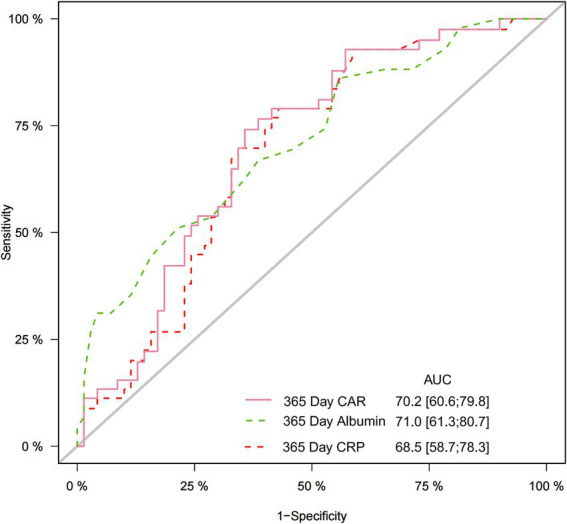
Receiver operating characteristic curves assessing the predictive ability of CAR, CRP and albumin. CAR, C-reactive protein/albumin ratio; CRP, C-reactive protein.

For 1-year mortality prediction (Figure 2), CAR achieved an AUC of 0.702 (95% CI 0.606–0.798), slightly below albumin (AUC 0.710) and above CRP (AUC 0.685). Incremental predictive analyses (Table 3) showed that albumin added modest but statistically significant predictive value over CAR (IDI 0.108, *p* = 0.012; NRI 0.217, *p* = 0.044); CRP did not provide significant incremental predictive value over CAR.

**Table 3:** Comparison of predictive performance for 1-year mortality between CAR, CRP and albumin.

Variable	AUC (95% CI)	IDI (95% CI)	*p*	NRI (95% CI)	*p*
CAR	0.702 (0.606 ~ 0.798)	Reference		Reference	
CRP	0.685 (0.587 ~ 0.783)	−0.010(−0.027 ~ 0.004)	0.200	−0.239(−0.377 ~ 0.248)	0.567
Albumin	0.710 (0.613 ~ 0.807)	0.108 (0.018 ~ 0.201)	0.012	0.217 (0.012 ~ 0.428)	0.044

CAR, C-reactive protein/albumin ratio; CRP, C-reactive protein; IDI: Integrated Discrimination Improvement; NRI: Net Reclassification Improvement; CI: confidence interval.

### 3.3 Subgroup analyses and sensitivity analyses

Subgroup interaction analyses (Supplementary Figures S1–S3) found no significant effect modification by age, sex, or BMI (all *P*-interaction > 0.05). Sensitivity analyses, using alternative CAR cutoff and alternative model specifications, yielded consistent results (Supplementary Tables S1, S2). E-value analyses (Supplementary Table S3) suggested moderate robustness of the CAR–mortality association (E-value 1.96); the albumin association was more robust (E-value 3.52), while the CRP association was less robust (E-value 1.45). As shown in Supplementary Table S4, in the high-albumin group, CAR was significantly associated with increased mortality (Model 2 HR 6.96, *p* = 0.037). CRP was also significantly associated with increased mortality (Model 2 HR 1.65, *p* = 0.041). Albumin itself showed no significant association. In the low-albumin group, albumin was significantly inversely associated with mortality (Model 2 HR 0.39, *p* = 0.002). CRP and CAR were not significantly associated with mortality and showed at most marginal significance.

## 4 Discussion

In this cohort of older Japanese patients with PSD, we observed that higher CAR and CRP were associated with increased mortality, whereas higher albumin was associated with lower mortality. Compared with the lowest tertile, the highest CAR tertile conferred a 3.77-fold increased mortality risk, and the highest CRP tertile a 3.92-fold increased risk; albumin ≥3.6 g/dL was associated with a 75% reduction in mortality risk. For 1-year mortality prediction, CAR had an AUC of 0.702, slightly lower than albumin (0.710) and higher than CRP (0.685). Stratified analyses show that the relationship between CAR and survival varies with baseline albumin levels. If patients have relatively good nutrition, the CRP component of CAR can detect higher inflammation. This detection helps predict worse survival. If patients have poor nutrition, measuring albumin directly may be more useful as a marker. Although several studies have evaluated the prognostic value of the CAR, important differences separate those studies from ours. For example, the meta-analysis by Yang et al. (14) confirmed an association between elevated CAR and poor outcomes but concentrated on acute stroke populations, did not address dysphagia, and their cohort was younger than ours. Uluç et al. (15) examined elderly patients in respiratory intensive care units (mean age 75.48 years), reflecting acute critical illness and acute inflammation. By contrast, our cohort had a mean age of 80.50 years; 86.5% had a CFS score ≥8, and 24.1% were concurrently diagnosed with aspiration pneumonia. These findings suggest that CAR in our study population reflects chronic inflammation and nutritional depletion rather than purely acute inflammatory responses. Guo et al. (16) studied Japanese elderly patients with dysphagia, but not specifically those with PSD. Our study therefore focuses on PSD patients and adjusted for stroke-related comorbidities such as ischemic heart disease and severe dementia.

After a stroke, dysphagia worsens the balance between nutrition and inflammation through multiple pathological mechanisms, which in turn affects clinical prognosis. This imbalance prolongs hospital stays and increases the economic burden on healthcare systems (17, 18). The relationship is bidirectional: low albumin weakens innate immune defenses, while elevated CRP reflects systemic inflammatory activation (19). In this context, the CAR is a practical composite sign that concurrently reflects inflammatory burden (CRP) and nutritional reserve (albumin) (20, 21). For clinicians, CAR could be a simple addition to risk assessment in older stroke patients–those with high CAR may benefit from prioritized multidisciplinary interventions to address nutrition and inflammation. When albumin is normal or near normal, an elevated CAR reflects inflammation. Clinicians should give this finding high priority. When albumin is low, low albumin indicates malnutrition. In those patients, prioritizing nutritional intervention may more directly improve prognosis than focusing only on inflammation. Additionally, there also is considerable potential to incorporate CAR into prognostic scores or to use it in clinical decision-making (like deciding on feeding strategies or goals of care).

To our knowledge, this is the first study in a dedicated PSD cohort demonstrating CAR’s prognostic value. Nevertheless, several limitations warrant consideration. First, because data were not collected prospectively, our analysis was restricted to the variables available in the dataset. We lacked information on stroke subtype; stroke severity (National Institutes of Health Stroke Scale); additional nutritional indicators (such as prealbumin); longitudinal measures such as trends in CAR and body weight; and objective swallowing assessments (the Wada drinking-water test and the Functional Oral Intake Scale). Second, as a small, retrospective secondary data analysis, the study is vulnerable to reverse causation, selection bias and potential overadjustment. Given an HR of 1.49 and an observed mortality of 45.9% over 4 years, the post-hoc statistical power was only ~62%. Although we adjusted for multiple confounders in multivariable models, residual confounding cannot be ruled out; to address this we fitted four supplementary models with alternative covariate sets and calculated E-values to assess the robustness of the primary associations to unmeasured confounding. Finally, the cohort comprised older Japanese patients with PSD from a single centre, limiting generalisability to other racial, ethnic or age groups. Determining optimal diagnostic or prognostic cut-offs requires prospective validation. Future studies should use large, multicentre prospective cohorts with standardised assessments to validate and extend these findings.

## 5 Conclusion

This cohort study indicated that a higher CAR is associated with increased mortality in older Japanese patients with PSD. CAR showed modest discriminative performance for 1-year mortality; albumin provided a small but statistically significant incremental improvement over CAR. Prospective, larger studies with richer covariate data are needed for validation.

## Data Availability

The datasets presented in this study can be found in online repositories. The names of the repository/repositories and accession number(s) can be found at: http://www.Datadryad.org/.
