# Supplementary material for: Association between C-reactive protein/albumin ratio and mortality in older Japanese patients with post-stroke dysphagia
Source: Front Neurol. 2025 Nov 21;16:1648517. doi: 10.3389/fneur.2025.1648517 (PMC12678102; doi:10.3389/fneur.2025.1648517)
Supplement: Supplementary file 1 [file Table_1.docx]

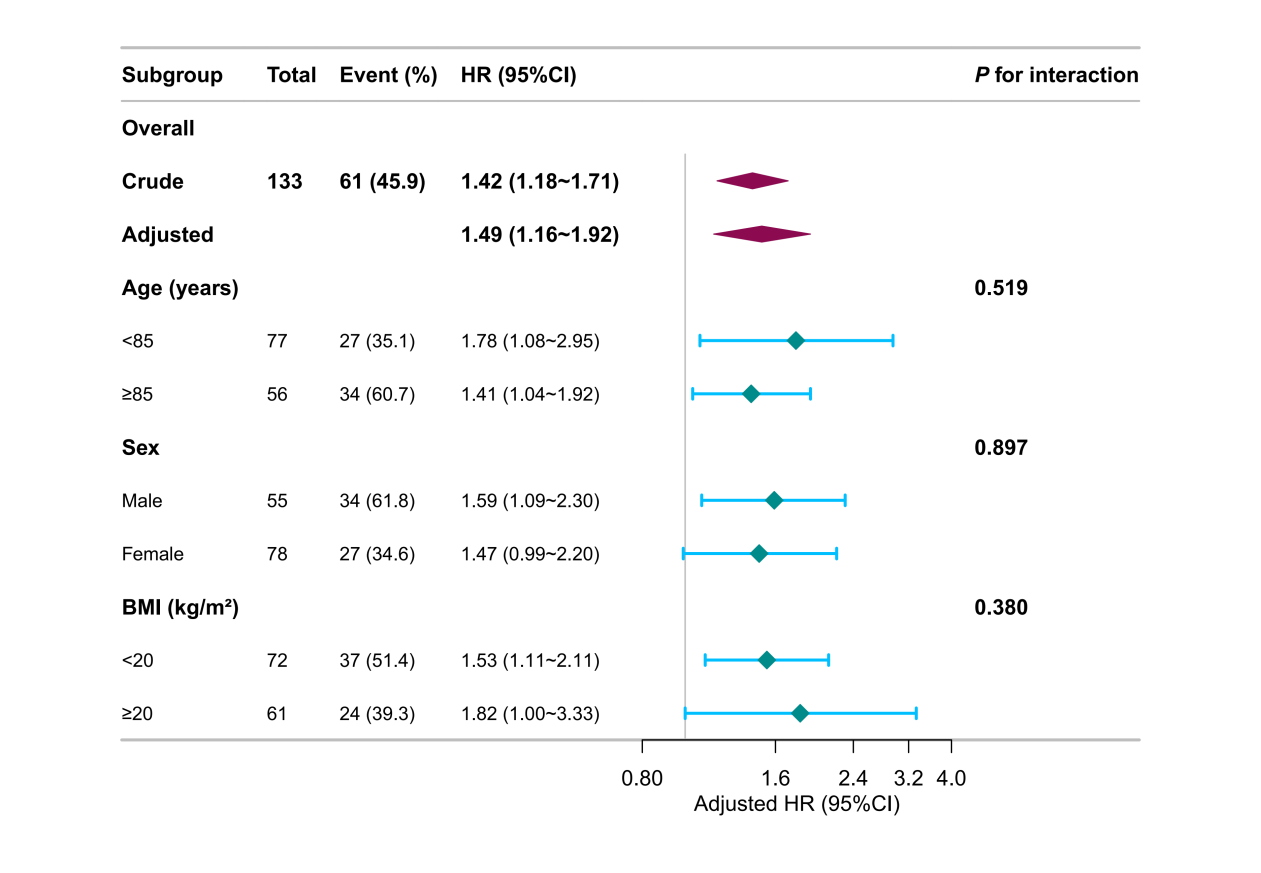


**Supplementary Figure S1.** Forest plots for survival in the different subgroups of CAR. Hazard ratios (HRs) were adjusted for age, sex, BMI, Clinical Frailty Scale scores, oral intake recovery and aspiration pneumonia. For subgroup analyses conducted within levels of a categorical variable, that variable was not included as a covariate in the subgroup-specific Cox models. BMI, body mass index; CAR, C-reactive protein/albumin ratio.


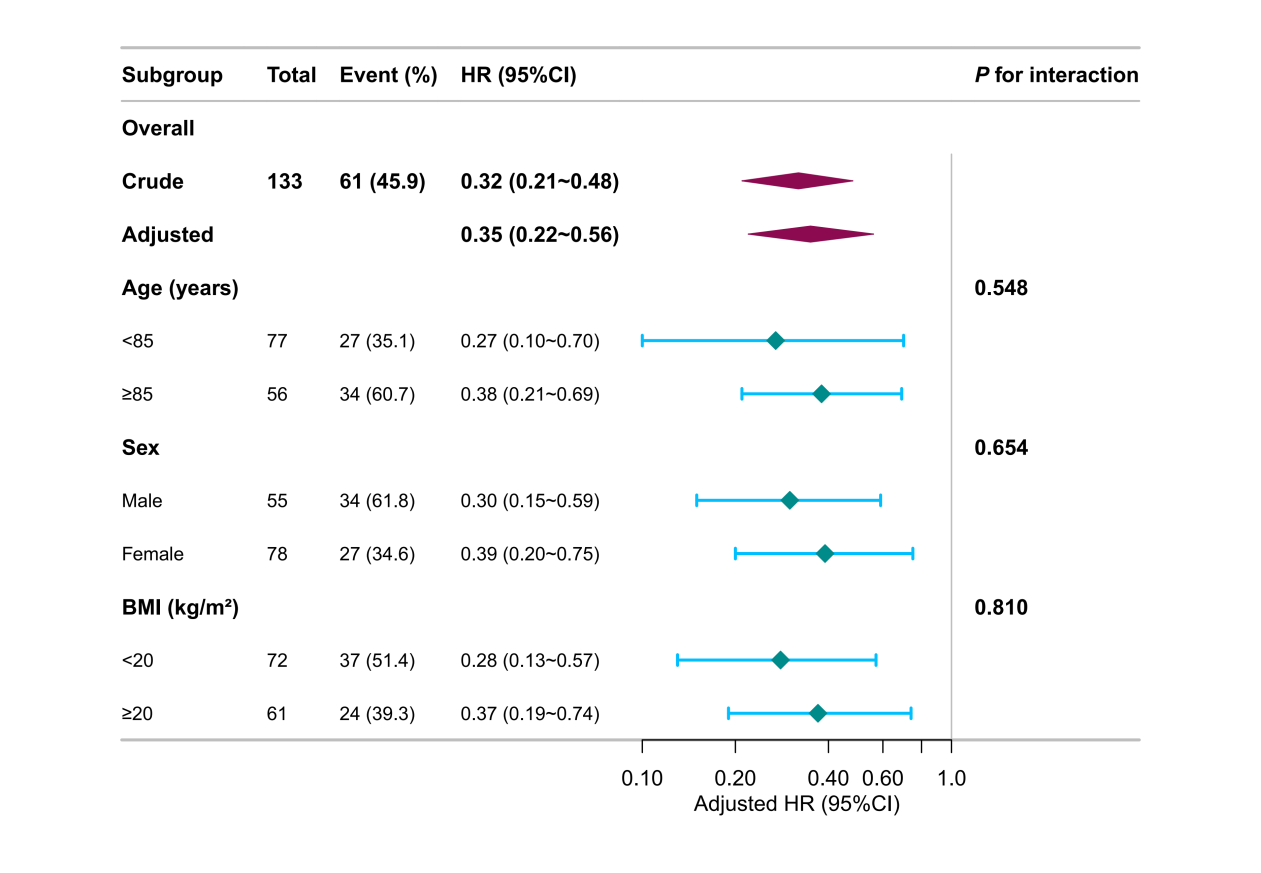


**Supplementary Figure S2.** Forest plots for survival in the different subgroups of albumin. Hazard ratios (HRs) were adjusted for age, sex, BMI, Clinical Frailty Scale scores, oral intake recovery and aspiration pneumonia. For subgroup analyses conducted within levels of a categorical variable, that variable was not included as a covariate in the subgroup-specific Cox models. BMI, body mass index.


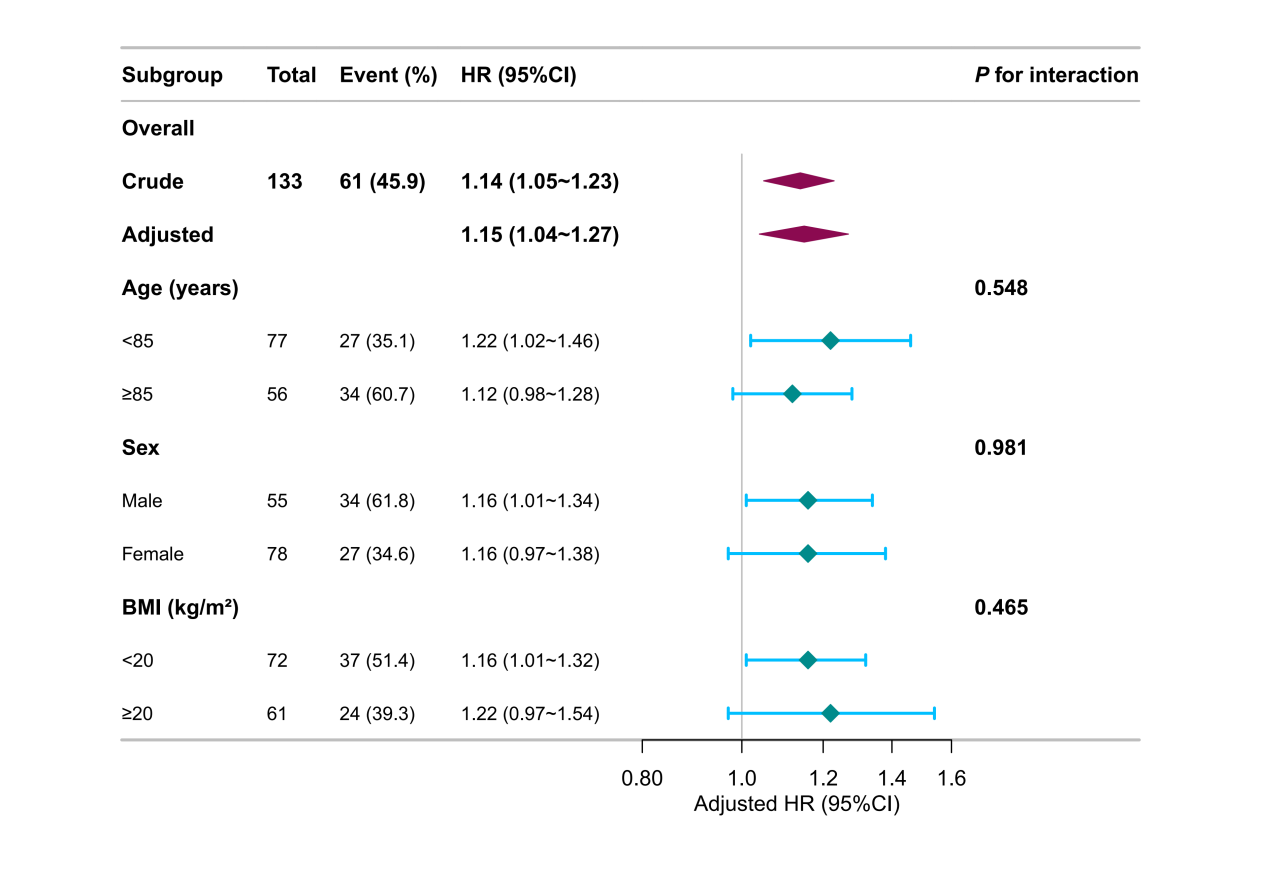


**Supplementary Figure S3.** Forest plots for survival in the different subgroups of CRP. Hazard ratios (HRs) were adjusted for age, sex, BMI, Clinical Frailty Scale scores, oral intake recovery and aspiration pneumonia. For subgroup analyses conducted within levels of a categorical variable, that variable was not included as a covariate in the subgroup-specific Cox models. BMI, body mass index; CRP, C-reactive protein.

| **Supplementary Table S1.** Association between CAR, CRP, albumin and mortality in different models. | | | | | | |
| --- | --- | --- | --- | --- | --- | --- |
| **Variable** | **Model 1** | | **Model 2** | | **Model 3** | |
|  | **HR (95%CI)** | ***P*** | **HR (95%CI)** | ***P*** | **HR (95%CI)** | ***P*** |
| CAR (T1, <0.240) | 1(Ref) |  | 1(Ref) |  | 1(Ref) |  |
| CAR (T2,≥0.240) | 3.95 (2.20~7.08) | <0.001 | 3.04 (1.66~5.57) | <0.001 | 3.13 (1.70~5.78) | <0.001 |
| CRP (T1, <0.83) | 1(Ref) |  | 1(Ref) |  | 1(Ref) |  |
| CRP (T2, ≥0.83) | 3.42 (1.93~6.06) | <0.001 | 2.69 (1.49~4.86) | 0.001 | 2.85 (1.57~5.19) | 0.001 |
| Albumin (T1, <3.3) | 1(Ref) |  | 1(Ref) |  | 1(Ref) |  |
| Albumin (T2, ≥3.3) | 0.34 (0.20~0.58) | <0.001 | 0.50 (0.29~0.86) | 0.013 | 0.46 (0.27~0.81) | 0.007 |
| Model 1: Non-adjusted; Model 2: Adjusted for age, sex and BMI; Model 3: Adjusted for age, sex, BMI, Clinical Frailty Scale scores, oral intake recovery and aspiration pneumonia. BMI, body mass index; CAR, C-reactive protein/albumin ratio. | | | | | | |

| **Supplementary Table S2.** Association between CAR, CRP, albumin and mortality in different models. | | | | | | | | |
| --- | --- | --- | --- | --- | --- | --- | --- | --- |
| **Variable** | **Model 1** | | **Model 2** | | **Model 3** | | **Model 4** | |
|  | **HR (95%CI)** | ***P*** | **HR (95%CI)** | ***P*** | **HR (95%CI)** | ***P*** | **HR (95%CI)** | ***P*** |
| CAR (continuous) | 1.54 (1.19~1.98) | 0.001 | 1.51 (1.16~1.97) | 0.002 | 1.44 (1.13~1.83) | 0.003 | 1.42 (1.11~1.82) | 0.006 |
| CAR (T1, <0.093) | 1(Ref) |  | 1(Ref) |  | 1(Ref) |  | 1(Ref) |  |
| CAR (T2,0.093~0.604) | 2.51 (1.07~5.87) | 0.034 | 2.34 (1.01~5.45) | 0.048 | 2.86 (1.20~6.80) | 0.017 | 2.49 (1.05~5.90) | 0.039 |
| CAR (T3, ≥0.604) | 3.48 (1.56~7.74) | 0.002 | 3.34 (1.49~7.49) | 0.003 | 2.94 (1.33~6.53) | 0.008 | 3.33 (1.48~7.51) | 0.004 |
| *P* for trend |  | 0.002 |  | 0.003 |  | 0.012 |  | 0.004 |
| CRP (continuous) | 1.16 (1.05~1.28) | 0.005 | 1.15 (1.03~1.28) | 0.009 | 1.14 (1.03~1.25) | 0.010 | 1.13 (1.03~1.25) | 0.012 |
| CRP (T1, <0.32) | 1(Ref) |  | 1(Ref) |  | 1(Ref) |  | 1(Ref) |  |
| CRP (T2,0.32~1.81) | 3.08 (1.34~7.08) | 0.008 | 3.03 (1.32~6.97) | 0.009 | 3.36 (1.46~7.75) | 0.004 | 2.87 (1.23~6.69) | 0.014 |
| CRP (T3, ≥1.81) | 3.63 (1.64~8.04) | 0.002 | 3.52 (1.57~7.87) | 0.002 | 3.33 (1.51~7.36) | 0.003 | 3.48 (1.54~7.90) | 0.003 |
| *P* for trend |  | 0.002 |  | 0.003 |  | 0.005 |  | 0.004 |
| Albumin (continuous) | 0.36 (0.23~0.58) | <0.001 | 0.38 (0.23~0.62) | <0.001 | 0.48 (0.30~0.78) | 0.003 | 0.50 (0.32~0.80) | 0.003 |
| Albumin (T1, <3.0) | 1(Ref) |  | 1(Ref) |  | 1(Ref) |  | 1(Ref) |  |
| Albumin (T2,3.0~3.6) | 0.40 (0.22~0.73) | 0.002 | 0.43 (0.23~0.78) | 0.006 | 0.44 (0.24~0.79) | 0.006 | 0.51 (0.28~0.92) | 0.026 |
| Albumin (T3, ≥3.6) | 0.28 (0.13~0.62) | 0.002 | 0.29 (0.13~0.66) | 0.003 | 0.37 (0.16~0.83) | 0.015 | 0.34 (0.15~0.77) | 0.010 |
| *P* for trend |  | 0.001 |  | 0.002 |  | 0.005 |  | 0.005 |
| Model 1: Adjusted for age, sex, Clinical Frailty Scale scores, oral intake recovery, severe dementia and aspiration pneumonia; Model 2: Adjusted for age, sex, Clinical Frailty Scale scores, oral intake recovery, severe dementia and BMI; Model 3: Adjusted for age, sex, Clinical Frailty Scale scores, oral intake recovery, percutaneous endoscopic gastrostomy and aspiration pneumonia. Model 4: Adjusted for age, sex, oral intake recovery, percutaneous endoscopic gastrostomy, severe dementia and ischemic heart disease. BMI, body mass index; CAR, C-reactive protein/albumin ratio; CRP, C-reactive protein. | | | | | | | | |
|  |  |  |  |  |  |  |  |  |
|  |  |  |  |  |  |  |  |  |

| **Supplementary Table S3.** E-value. | | | |
| --- | --- | --- | --- |
|  | **CAR** | **Albumin** | **CRP** |
| HR  (95%CI) | 1.49  (1.16~1.92) | 0.35  (0.22~0.56) | 1.15  (1.04~1.27) |
| E-value | 1.96 | 3.52 | 1.45 |
| Hazard ratios (HRs) were adjusted for age, sex, BMI, Clinical Frailty Scale scores, oral intake recovery and aspiration pneumonia. BMI, body mass index; CAR, C-reactive protein/albumin ratio; CRP, C-reactive protein. | | | |

| **Supplementary Table S4.** Association between CAR, CRP, albumin and mortality among different albumin level in different models. | | | | |
| --- | --- | --- | --- | --- |
| **Variable** | **Model 1** | | **Model 2** | |
|  | **HR (95%CI)** | ***P*** | **HR (95%CI)** | ***P*** |
| **Albumin≥3.6g/dl** | | | | |
| CAR (continuous) | 17.51 (2.97~103.19) | **0.002** | 6.96 (1.13~42.95) | **0.037** |
| CRP (continuous) | 2.12 (1.32~3.40) | **0.002** | 1.65 (1.02~2.67) | **0.041** |
| Albumin (continuous) | 0.08 (0.00~1.90) | 0.117 | 0.20 (0.01~7.60) | 0.383 |
| **Albumin<3.6g/dl** | | | | |
| CAR (continuous) | 1.20 (0.96~1.50) | 0.114 | 1.27 (1.00~1.63) | 0.051 |
| CRP (continuous) | 1.06 (0.96~1.16) | 0.238 | 1.08 (0.98~1.19) | 0.131 |
| Albumin (continuous) | 0.41 (0.23~0.74) | **0.003** | 0.39 (0.22~0.70) | **0.002** |
| Model 1: Non-adjusted; Model 2: Adjusted for age, sex and BMI. BMI, body mass index; CAR, C-reactive protein/albumin ratio; CRP, C-reactive protein. | | | | |
|  |  |  |  |  |
